# The dilemma of using racial and ethnic categories in biomedical research

**DOI:** 10.1186/s12874-026-02917-x

**Published:** 2026-07-08

**Authors:** A S Kidane Gebremeskel, K A C Meeks, M H Cnossen, A M’charek, A W Rijneveld

**Affiliations:** 1 Dep. of Pediatrics, Pediatric Hematology, Erasmus Medical Center, Dr. Molewaterplein 40, Rotterdam, South Holland 3015 GD the Netherlands; 2 Center for Research on Genomics and Global Health, National Human Genome Research Institute, National Institutes of Health, The United States of America, 12 South Drive Bldg 12A ste 1025, Bethesda, MD 20892-5611 USA; 3 Anthropology, University of Amsterdam, Nieuwe Achtergracht 166, Amsterdam, North Holland 1018WV the Netherlands; 4 Department of Hematology, Erasmus University Medical Center, Dr. Molewaterplein 40, Rotterdam, South Holland 3015 GD the Netherlands

**Keywords:** Sickle cell disease, Ethnicity, Race, Ethno-racial categories, Confounding factors

## Abstract

Ethno-racial categories (ERCs) are inconsistently operationalized in biomedical research, with challenges in incorporating multiracial subjects and reliance on proxies like country of birth. We emphasize the need for transparency in ERC data collection and propose peer reviewer guidelines to standardize and critically evaluate ERC usage in studies.

## Introduction

In biomedical research, race and ethnicity are frequently operationalized as confounders [1]. Ethno-racial categories (ERCs) reflect assumptions of a higher within- group than between-group homogeneity. In practice, however, ERCs include a wide range of biological and social diversity. Moreover, racialization processes vary across time, geography, and sociopolitical contexts, making race inherently unstable as a categorization target. Using ERCs incautiously has many undesirable consequences, such as further enforcing notions of biological and social essentialism. This may compromise the integrity of research outcomes and pose risks to the quality of health care delivery.

In our recent analysis of sickle cell disease (SCD) research articles, we showed that the contextual characteristics of ERCs are often given insufficient consideration. Not only was the rationale behind the operationalization of ERCs rarely mentioned, the methodology through which ERCs were ascertained, was often missing. Furthermore, in 28% (83/298) of articles that were ERC-adjusted, a specific ethnic or racial identifier was not referenced [2]. Similar patterns of incomplete reporting and lack of methodological transparency in ERC operationalization have been documented across general epidemiological and clinical journals [3–5]. This gives the impression that the application of ERCs in biomedicine lacks a clear and systematic approach. Incomplete reporting on ERCs in biomedical studies can further strengthen the notion of ERCs being self-evident concepts in biomedical research.

In this paper, we use the term “ethno racial categories” or ERCs to refer to groups of individuals with a common geographical ancestry and/or having a similar sociocultural background. Ethno-racial categories may include nationalities (Surinamese and Welsh), races or ethnicities (Black or Hispanic Americans), and communities with a common sociocultural background that are not demarcated by a nation state (Kurdish, Oromo, Amazigh).

Because ERCs are aggregate constructs, they encompass substantial biological and social heterogeneity. This heterogeneity is often insufficiently considered when ERCs are used in biomedical research, either as descriptive labels or analytic variables. What is considered an ERC, varies across time, place and research context, underscoring that ERCs are social constructs that are continuously shaped by human interactions and practical needs rather than fixed definitions.

This Comment seeks to raise awareness among biomedical researchers. We also hope to contribute to a debate on the misconceived nature of ERCs when operationalized as confounders. Furthermore, we mention additional recommendations for responsible use of ethnic and racial categories in biomedical research.

## Dilemmas concerning the use of ERCs as confounders

Confounding is a major methodological challenge in biomedical research that can result in distorted study findings. A confounder is a variable that is associated with both the exposure and outcome of interest in a study. We discuss three confounder-adjustment techniques and the associated dilemmas when used in relation to ERCs.

### Matching

The first method is case-control matching. Matching ensures an even distribution of a confounder across groups; a simple, cost-effective approach to correct for confounders. On the other hand, matching could introduce selection bias and obscure the strength of a hypothesized confounder. In our review,^2^ one cross-national study compared SCD patients living in France (originating from West or Central Africa or the West Indies) with healthy controls living in Congo to investigate autoantibody prevalence [2]. The researchers matched on geographic origin, with some overlap between Central African patients and Congolese controls. However, since autoimmune responses can be influenced by environmental factors and infectious disease exposures, differences in autoantibody prevalence may reflect contextual factors related to living in France versus in Congo rather than SCD-related immune dysregulation alone [6]. Matching on ERCs poses a risk of reinforcing the unverified relevance of race and ethnicity. Therefore, careful consideration and a high level of confidence are required when choosing matching as a confounder-adjustment technique, especially in a research climate where ERCs are often presented as seemingly self-evident concepts.

### Restriction of study enrollment

Restriction of enrollment is another commonly used method of confounder adjustment. Ethnic minorities in Western countries or non-European study populations are perceived as “deviant” from the “normal” European cases. This view has led to exclusion and therefore underrepresentation of non-European-ancestry individuals in genome-wide association studies (GWAS) [7]. GWAS studies identify candidate genetic variants which may play a role in a particular disease. The presence of genetic variations that are correlated with certain diseases and risk factors, can be aggregated into polygenic risk scores, which are intended to improve biomedical outcomes. However, because of this Eurocentric bias in the GWAS studies, these polygenic risk scores translate poorly from Europeans to non-Europeans, thus limiting possibilities for individuals with non-European genetic ancestry to benefit from developments such as personalized medicine [8]. This contributes to a further increase in health differences between minority and non-minority populations in Western societies.

### Stratification

Stratification is a method that aims to produce homogenous groups based on certain characteristics. This is currently the preferred method for genomics research. By contrast, trans-ancestry genomics studies such as GWAS analyze participants of any genetic ancestry together, thereby often obscuring findings in non-European-ancestry groups because of the stark imbalance in the number of European-ancestry samples versus non-European samples. In this approach, important ancestry-specific variants may be missed.

### The impossible task of categorizing people

In order to use ERCs in a specific confounding method as discussed above, study participants have to be categorized. Genetic ancestry is, however, a continuum and making categories out of this continuum is like “slicing soup;” no matter where you cut, the soup stays mixed. At the same time, there is a clear need to increase diversity in biomedical, epidemiological, and genomics studies. In non-genomics studies, the self-report method is most commonly used to classify people into ERCs. Also, the use of labels from pre-existing datasets is often seen [2]. 

By definition, ERCs lack universality. Rather than facing the inherent contextuality of ERCs as a drawback, researchers should embrace this characteristic of ERCs by providing clear documentation.

### Instability of the self-report method

Race and/or ethnicity of study participants is often registered through self-report. In our analysis in SCD research, 9% (27/298) of all selected articles indicated using self-report [2]. Self-report creates an unstable foundation as ERCs are non-fixed categories that are also influenced by shifts in sociopolitical context, across time and geography. Within the UK census between 1991 and 2001, 23% of both the “Caribbean” and “African” groups switched categories while those identifying as White remained stable [9]. Similarly, “Mexican” was removed from the American census as a race in 1930 in part due to lobbying by Mexican-American interest groups, with “Hispanic” re-appearing as an ethnic category in 1970 [10]. Especially when ethno-racial self-identification is restricted by multiple-choice answer options in a survey, bias is introduced. Moreover, technologies such as genetic ancestry testing do not reliably predict racial or ethnic self-identification. Although genetic ancestry testing [11] has contributed to an increasing ‘geneticization’ of identity, individuals actively interpret ancestry results in light of their social contexts and identity goals rather than adopting them uncritically [12, 13]. This presents a fundamental challenge to treating ERCs as stable or objectively measurable variables in biomedical research.

### Individuals identifying themselves as a mix of ERCs

Individuals are increasingly identifying as multi-ethnic or multiracial and that adds a layer of complexity to the classification conundrum. The 2011 England and Wales Census, saw an increase of 79% of individuals identifying as mixed/multiracial in one decade; twice as fast as in the United States [14]. This trend further challenges the validity of current ERC classifications. However, in only 2% (23/1,085) of all domestic research articles from our analysis in SCD mixed-race/multi-ethnic individuals were mentioned, which might be an underestimation of the true number of mixed research participants since an estimated 61% of people with mixed-race ancestry do not mark themselves as multiracial in surveys as described in a U.S. survey of 1,555 multiracial individuals aged 18 and above [15, 16]. 

### Processing multiracial individuals as monoracial through “race bridging”

Multiple-race responses have been introduced by the 2000 U.S. Census and 2001 UK Census surveys. However, when data from large-scale databases are adopted for biomedical research, they create methodological challenges for researchers [17]. This often leads researchers to restrict enrollment of multiracial individuals, categorize them as a single, residual category or group them with a related single racial category [18, 19]. Categorizing multiple-race responses into a single-race category is known as race bridging for which specific algorithms are in place [19]. These algorithms rely on statistical models derived from multiracial respondents in prior surveys selected a single “primary race” when forced to choose. Consequently, the resulting classifications reflect patterns of self-identification shaped by racialization processes, identity salience, and contextual pressures inherent to constrained classification systems [20]. 

This trend to diminish multiracial self-identification is in contrast with the demographic increase in individuals identified with multiple races or ethnicities. This, in turn, presents the world as less complicated and diverse than it in reality is.

### Variations and inconsistencies in ERC taxonomies and datasets

In biomedical research, pre-existing datasets, for instance rendered by governmental agencies, are often integrated with new data of study participants to generate a comprehensive research dataset. However, these datasets are often not harmonized in ERCs used and are further complicated by before-mentioned challenges in classifying mixed individuals.

In a survey sent to public health officials in the New England area of the U.S., researchers found inconsistencies across 169 public health data systems; 33% (*n* = 57) of these systems did not comply with the U.S. federal standard and only provided a race field without ethnicity fields. The ethno-racial labels used were not fully comparable [21]. Moreover, several surveys had different ways of classifying certain minority groups under standardized ERCs. This was the case for “Brazilians,” which do not comfortably fit the standard ERCs from the U.S. Office of Management and Budget (OMB) labels such as “Hispanic/Latino” ethnicity [21]. 

These inconsistencies in the registration practices of ERCs are also impacted by existing discrepancies between standardized classifications of ERCs and self-reported ERCs in health care systems [22]. This discordance is further increased by individuals who identify themselves as “mixed.” Comparing Hospital Episode Statistics (HES) data with NHS data in the UK, in which ethnicity was self-assigned, showed that HES-recorded ethnicity was found to be discordant for 74% of records for the “Mixed” group [23]. Furthermore, mixed “White and Black African” patients who were admitted multiple times between 2003 and 2006 only had a 70% chance that they would be consistently coded into the same ERC upon recurring admission, compared to 95% of “White British” patients [24]. 

### Inferring race and ethnicity

Biomedical research often uses demographic data from datasets created by governmental agencies. In these large datasets, self-reported race and ethnicity are often re-categorized into large, standardized racial categories. Furthermore, indirect estimation methods are also used when the data are missing to infer the race of individuals [25]. This algorithm is issued by the Bayesian Improved Surname and Geocoding (BISG) to determine the ERC of Medicare beneficiaries [26]. Here, statistical methods are used to combine U.S. Census information on race and ethnicity by both surname and location of residence to produce a set of ERC categories per person, each with a probability of belonging to that ERC [27, 28]. Self-reported ERCs can significantly deviate from assigned ERCs in large datasets, depending on the specific ERC. One study found that over 80% of self-reported American Indian/Alaskan Natives in 24 USA states and Puerto Rico were misclassified in pre-labeled datasets that determined race and ethnicity through various ways, including imputation algorithms [29]. Misclassification can substantially bias health inequality estimates. For example, poor ethnicity data quality in Scottish NHS records masked a 68% elevated COVID-19 risk among White Gypsy/Travellers and underestimated risks for Bangladeshi and Arab groups [30]. Beyond obscuring true disparities, these data quality issues limit statistical power and can lead to inappropriate aggregation of heterogeneous ethnic groups, ultimately threatening the ability to effectively monitor and address ethnic health inequalities [30, 31]. 

### Country of birth: a popular proxy for ERCs

In several countries, race and ethnicity data are indirectly measured using proxies. In the Netherlands and other European countries, country of birth has long been used as a proxy for ethnicity in health policy and research [32]. Advantages of this method are that country of birth and its derivative, country of origin is measurable and immutable, allowing for easy comparison across studies. For instance, Dutch citizens who were either born or had a parent born outside the Netherlands were divided into a Western or non-Western migration background. This ontology made it possible to indirectly discern Dutch citizens of color. However, this method also has its limitations. Third generations and beyond with a migration background become administratively undetectable as citizens of color, since both parents are born in the Netherlands. Furthermore, the Western/non-Western dichotomy, now abandoned, created paradoxical classifications: Dutch-descended Afrikaners from South Africa would be categorized as non-Western while persons with Japanese and Indonesian backgrounds were classified as Western based on their socioeconomic position and historical ties to the Netherlands. Health researchers using this measure have encouraged collecting more granular environmental determinants such as geographic origin, language and self-identified ethnic group. Country of birth as ethnicity-proxy is often reduced into a dichotomous index, with or without a non-Western migration background [33]. Recently, there have been changes in the taxonomy of migration background; the division between Western versus non-Western has been removed [34]. 

There are various methods for ethnic and racial classification. When conducting retrospective research, research teams often work with pre-collected data. It is therefore pivotal to document clearly which categorization process was used. This makes it possible to infer potential repercussions for specific research goals.

### Dangers of using ERCs in biomedical research

Whether ERCs are relevant to account for in biomedical research, depends on the research question. ERCs are social constructs, rather than naturally created boundaries that accurately reflect human biological variation. On one hand, ERCs can be an initial step to explore both biological and environmental variations within a group. On the other hand, biological and social determinism are relevant risks when operationalizing ERCs in biomedical research. This makes ERCs a double-edged sword that needs to be handled carefully. The improper use of ERCs has direct and indirect negative consequences. One well-known example of race-based medicine is the correction factor for calculating glomerular filtration rate. This algorithm leads to an overestimation of eGFR followed by undertreatment for populations racialized as black and has therefore in 2021 been replaced by a “race-free” algorithm [35]. An example of social essentialism is ascribing health behaviors to one’s culture, therefore approaching culture as a static framework. This, for example, results in stereotypes of non-Western patients entering medical curricula [36]. These cultural stereotypes reinforce the idea of a non-ethnic Dutch, or “white” patient as outside the implicit normative cultural standard [36]. This is in contrast with research involving second- and third-generation immigrants in Europe who are often well integrated with many different cultural backgrounds [37]. Aside from direct consequences for patients of color, the reification of race and ethnicity also has indirect consequences for this group and society in general. Differences between racial and ethnic groups should be regarded as a starting point into further scientific inquiry. For example, limiting GWAS studies to participants with European ancestry, decreases the yield of these studies for other groups, and prevents a global understanding of health and disease [7]. 

### Moving forward: additional recommendations

Recently, author guidelines have been published by various clinical journals [38]. Most of them acknowledge that race and ethnicity are social constructs and contextualization is needed as well as active engagement from studied communities in the research process. Moreover, the method through which ERCs are determined and limitations introduced when using this method should be stated. However, several uncertainties concerning the use of ERCs are not fully established and therefore prone to inconsistencies. To guide this process, we recommend establishing peer-reviewers’ guidelines surrounding the operationalization of race and ethnicity in biomedical research. This ensures adherence to the author’s guidelines and encourages reviewers to maintain a critical perspective throughout their evaluation of a manuscript.

Furthermore, we pose that more research is needed on the effects of racism on health, which is often an environmental stressor that might overlap with self-identified or perceived racial/ethnic origin [39]. Currently, the (potentially) numerous pathways through which racism negatively impacts health are understudied. A review investigating over 200,000 articles published over the past 30 years in impactful biomedical journals (e.g., BMJ, NEJM, JAMA, and the Lancet) stated that less than 0.1% of articles included the word racism anywhere in the text [40]. 

Recently, a National Academies of Sciences Engineering and Medicine (NASEM) committee, recommended narrower application of race and ethnicity in genomics and biomedical research. In genomics studies, they recommended using ethnic and racial categories (ERCs) only as proxies for environmental effects [41, 42]. For broader biomedical research, they recommend scrutinizing ERC use at every research stage, disclosing limitations using secondary datasets, investigating underlying mechanisms beyond race and ethnicity, and ensuring inclusion and equity throughout [42]. 

## Conclusion

This Comment provides a perspective on the instability of racial and ethnic categories, and the dilemmas that engage when operationalizing them as confounders. The labels that are assigned to individuals at the start of the research process are not always the labels used in the final manuscript of the study. When deciding to operationalize ERCs as confounders, it is the responsibility of the research team to assess the need of ERCs as confounders and place them in the context of the study.

When ERCs are considered important confounding factors in a specific research project, they should be contextualized. Solely operationalizing ERCs in biomedical research without contextualization further contributes to the reification of ERC as a biological category. By definition, an integrative analysis is needed, considering the many different mechanisms through which human difference is established.

## Data Availability

No datasets were generated or analysed during the current study.
